# Analysis of evolutionary relationships provides new clues to the origins of weedy rice

**DOI:** 10.1002/ece3.5948

**Published:** 2019-12-20

**Authors:** Bing Han, Xiaoding Ma, Di Cui, Yanjie Wang, Leiyue Geng, Guilan Cao, Hui Zhang, Hee‐Jong Koh, Longzhi Han

**Affiliations:** ^1^ Institute of Crop Sciences Chinese Academy of Agricultural Sciences Beijing China; ^2^ Department of Plant Science, Plant Genomics and Breeding Institute of Agriculture and Life Science Seoul National University Seoul Korea

**Keywords:** comparative transcriptome analysis, genetic diversity, haplotype analysis, weedy rice

## Abstract

Weedy rice (WR) (*Oryza sativa f. spontanea*) is considered to be a pest in modern rice production systems because it competes for resources, has poor yield characteristics, and subsequently has a negative effect on rice grain yield. The evolutionary relationships among WR, landrace rice (LR), improved rice (IR) cultivars, and wild rice are largely unknown. In this study, we conducted a population genetic analysis based on neutral markers and gene haplotypes in 524 rice accessions and a comparative transcriptomic analysis using 15 representative samples. The results showed that WR populations have the highest level of genetic diversity (*H*_e_ = 0.8386) and can be divided into two groups (*japonica*‐type and *indica*‐type). The *japonica*‐type WR accessions from Heilongjiang province (HLJ), Jilin province (JL), Liaoning province (LN), and NX provinces clustered with the landraces grown in these same provinces. The *indica*‐types from Jiangsu province (JS) also clustered with the *indica*‐type landraces from JS province. Comparative transcriptome analysis of WR‚ IR and LR from HLJ, JL, and LN provinces showed that the WR still clustered with the LR, and that the IR lines comprise a single population. Thirty‐two differentially expressed genes were shared by the IR and LR groups as well as between the IR and WR groups. Using Gene ontology (GO) analysis, we identified 19 shared GO terms in the IR and LR groups as well as between the IR and WR groups. Our results suggest that WR populations in China have diverse origins, and comparative transcriptome analysis of different types of rice from HLJ, JL, and LN provinces suggests that IR populations have become a end point in the evolution of WR, which provides a new perspective for the study of WR origins and lays a solid foundation for rice breeding.

## Graphical abstract

In this study, we conducted a population genetic analysis based on neutral markers and gene haplotypes in 524 rice materials and then conducted a comparative transcriptome analysis in 15 representative samples. Our results suggest that weedy rice (WR) populations in China have diverse origins, and comparative transcriptome analysis of different types of rice from Heilongjiang province, Jilin province, Liaoning province suggests that improved rice populations have become a medium or end point in the evolution of WR, which provides a new perspective for the study of WR origins and lays a solid foundation for rice breeding.

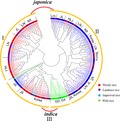

## 1 INTRODUCTION

Rice (*Oryza sativa* L.) is one of the most important food crops worldwide because it provides daily sustenance for one‐third of the world's population. Weedy rice (WR) commonly grows as a weed in paddy fields and has phenotypes that are intermediate between cultivated rice (*O. sativa*) and wild rice (*O. rufipogon*). In a landmark study, Suh, Sato, & Morishima (1997) characterized WR and classified it into two types that correspond to the two major rice subspecies, *indica* and *japonica*, and then divided it into four forms (I, II, III, and IV), categorizing the similarities between cultivated and wild rice. Group I is mainly distributed in temperate countries and belongs to the *indica*‐type, similar to agricultural cultivars. Group II also belongs to the *indica*‐type and is similar to wild rice forms which are distributed in tropical countries. Group III is primarily distributed in Bhutan and Korea and belongs to the *japonic*a‐type and is similar to other cultivars. Group IV belongs to the *japonic*a‐type and is similar to wild rice found in China and Korea (Suh et al., 1997). Kawasaki, Imai, Ushiki, Ishii, & Ishikawa (2009) studied WR in Okayama, Japan and found that the *japonica*‐types shared the same haplotypes with local cultivated varieties, and the *indica*‐types had two haplotypes in common with the forage varieties, one of which was a deletion of *OsGA20ox2*, a semi‐dwarf allele. Akasaka, Ushiki, Iwata, Ishikawa, & Ishii (2009) concluded that WR from Okayama, Japan originated from cultivated rice varieties.

In recent years, the possible origins of WR have received considerable attention around the world. Genetic studies have indicated that WR has diverse origins, and that it evolved independently from domesticated rice or its wild relatives in different regions. Many studies of WR are mainly focused on those geographical regions where no reproductively compatible wild rice species occur, such as North America, North China, and Korea. Sun et al. (2013) found that introgression and selection from cultivated rice contributed to the genome of WR in northern China, and Sun et al. (2019) showed that WR from high latitude regions in Asia possesses many unselected genomic characteristics. The study of He, Kim, and Park (2017) indicated that Korean WR originated from hybridization of modern *indica*/*indica* or *japonica/japonica* varieties rather than from wild rice, while Li, Li, Jia, Caicedo, and Olsen (2017) showed that WR has evolved through de‐domestication from cultivated varieties in America. However, in Southeast Asia and South China, WR and wild rice occur sympatrically. The origins of WR in these regions are more complicated than it is in places where no reproductively compatible wild rice species exist. Wild rice and elite cultivars shaped agricultural WR evolution in Southeast Asia (Song, Chuah, Tam, & Olsen, 2014), while Qiu et al. (2014) suggest that WR originated from hybridization of domesticated *indica*/*japonica* varieties based on a case study from southern China. There is also evidence to show that genomic variation is related to local adaptation of WR during de‐domestication (Qiu et al., 2017). Thus, the origins of WR are complex and are the source of some controversy at present. In general, there are two primary hypotheses regarding the origins of WR in regions where there is no sympatric occurrence of WR and wild rice: (a) de‐domestication from cultivated varieties and (b) gene introgression from cultivated varieties. In addition to these two hypotheses, in regions where WR and wild rice both occur, gene introgression from wild rice may also be a factor in the origin of WR.

In China, rice domestication was a long and complex process that required movement away from the common ancestor to *O*. *rufipogon*, from *O*. *rufipogon* to landrace rice (LR) varieties, and then from landraces to improved rice (IR) varieties. According to the domestication history of rice, prior to the 1950s, the most well‐suited varieties in the rice‐growing regions of Heilongjiang (HLJ), Jilin (JL), Liaoning (LN), Ningxia (NX), and Jiangsu (JS) provinces of China were local landraces or introduced varieties. The first Green Revolution had an important effect on rice domestication, especially in China, where natural dwarf mutants were selected and bred from the local high‐stature landraces grown at that time. A series of exceptional dwarf varieties were developed through hybridization with natural dwarf mutants. This is the only time in the history of rice domestication that a superior landrace was cultivated at a large scale for the purposes of rice breeding. This also presented a unique opportunity to utilize a gene from a landrace in the IR genome and enabled the transfer of landrace genes into the modern IR genome. Therefore, some LR can be regarded as the short‐stage domestication ancestors of IR (Mann, 1997). After the 1980s, many rice varieties with desirable improved characteristics were produced and commercialized. During this period, landraces were eliminated from rice production systems in China. Between 1980 and the 2010s, LR disappeared from China and was replaced by IR varieties. In our study, IR samples from 1980 to the 2010s and WR lines were collected from IR fields and from other fields in HLJ, JL, LN, NX, and JS provinces (Luo et al., 2014; Wang et al.,^2010^; Min, Shen, & Xiong, 1996). Wild rice cannot be grown in HLJ, JL, and LN provinces due to unsuitable environmental conditions. For this reason, genes from wild rice cannot have been transferred into modern IR varieties via natural hybridization. Furthermore, in HLJ, JL, and LN provinces, LR populations have disappeared from the rice fields over the last 30 years, which means that the genomes of WR in these provinces cannot have experienced gene introgression from local LR. Therefore, it is our hypothesis that the origin of WR in China, specifically in LN, JL, and HLJ provinces, has a connection with modern IR.

In previously published studies, researchers focused on the evolutionary relationships between WR and cultivated rice which has limited our understanding of the relationships among these rice groups. In our study, we found that cultivated rice lines are clearly divided into landraces and improved cultivars, allowing for a more precise determination of the evolutionary relationships between WR and landraces or IR varieties. Our results will provide a new perspective on the origins of WR and will allow strategies for weed control and management in rice paddy fields.

## 2 MATERIALS AND METHODS

### 2.1 Plant materials, DNA extraction, and SSR molecular marker assays

In total, 524 rice accessions, including 136 WR samples, 161 LR samples, and 185 IR samples from Heilongjiang (HLJ), Jilin (JL), Liaoning (LN), Ningxia (NX), and Jiangsu (JS) provinces and Korea, as well as 42 wild rice varieties from Guangxi (GX) and Guangdong (GD) provinces, were planted in an experimental field in Hainan Province, China, in January 2016 (Figure S1 see Dryad upload). Leaf tissues from all plants were obtained for DNA extraction in March 2016. Two parts of each leaf were sampled: one part was used for SSR marker detection by composite sampling, while the other part was used for haplotype analysis of individual plants. DNA was extracted using the modified CTAB method (Doyle & Dickson, 1987). DNA concentration was determined using a Nano Drop 2000 spectrophotometer (Thermo Fisher Scientific), and DNA integrity was checked by electrophoresis on 1% agarose gels.

Fluorescently labeled oligonucleotide primers for 36 SSR markers (Table S1 see Dryad upload) were prepared by Biotechnology Company. PCR amplifications were carried out using the following thermal cycling program: a predenaturation step at 94°C for 5 min, followed by 35 cycles of 94°C for 30 s, 50–60°C for 30 s, and 72°C for 1 min, with a final extension at 72°C for 5 min. PCR products were sequenced by TSINGKE on a 3730XL DNA Sequencer (Applied Biosystems Inc.). Fragment lengths were analyzed using Gene Marker V1.6 (Soft Gene), and the data were retained for later analysis.

**Figure 1:**
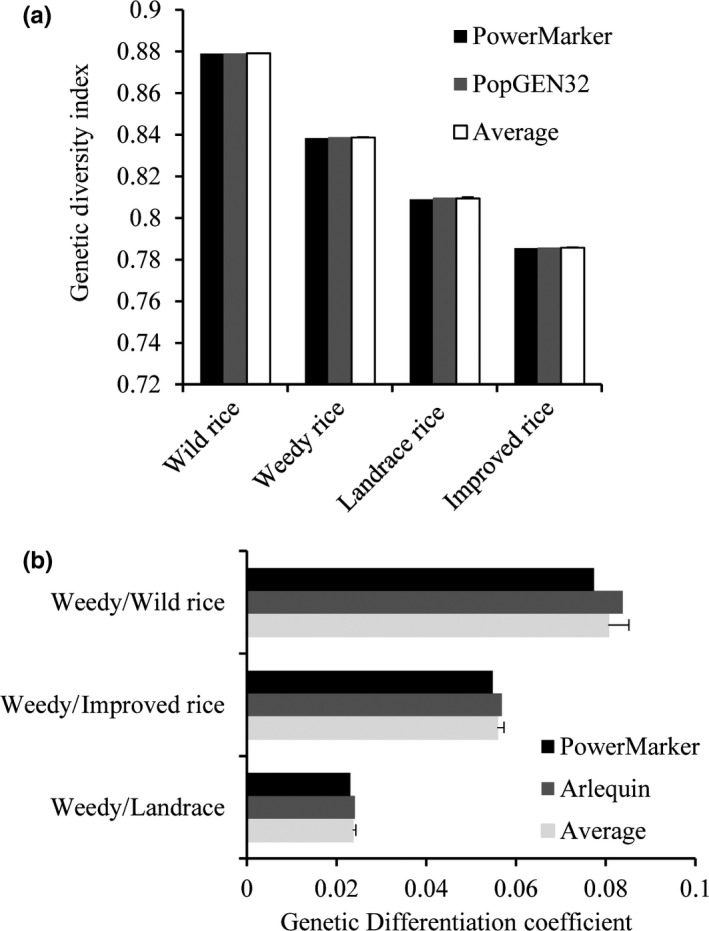
Genetic diversity analysis of the 524 genotypes based on 36 pairs SSR markers. (a) Genetic diversity index of weedy rice (WR) and other population. (b) Genetic differentiation coefficient among population. Power Marker, PopGEN32, and Arlequin are the different analysis software

We estimated the genetic diversity of the 524 samples from the four populations (WR, LR, IR, and wild rice) using data from 36 SSR loci. The observed number of alleles (*N*_a_), effective number of alleles (*N*_e_), Shannon's Information index (*I*), and Nei's expected heterozygosity (*H*_e_) were calculated with PopGEN32 software (http://cc.oulu.fi/~jaspi/popgen/popdown.htm). Genetic diversity, heterozygosity, PIC, and the inbreeding coefficient (*f*) were calculated with Power Marker V3.25 (Liu & Muse,^2005^). In addition, genetic diversity coefficients (*F*_ST_) between populations were calculated using two methods implemented in Arlequin ver 3.1 (http://cmpg.unibe.Ch/software/arlequin3/) and Power Marker V3.25 (Liu & Muse, 2005). In this study, the mean values of *F*_ST_ from Arlequin ver 3.1 and Power Marker V3.25 (Figure 1) were used to evaluate the degree of genetic differentiation among the four rice populations (WR, LR, IR, and wild rice).

The DNA sequences of parts of six rice genes (*Hd1*, *D3*, *DL*, *OsMYB2P*, *Pita*, and *OsBADH2*) were obtained from the NCBI database, and oligonucleotide primers were designed using primer5. PCR amplification was carried out using the following thermal cycling program: predenaturation at 94° for 5 min, followed by 35 cycles of 94°C for 30 s, 55–62°C for 30 s, and 72°C for 1 min, with a final extension at 72°C for 10 min. PCR products were Sanger sequenced, and data were retained for later analyses.

### 2.2 Population structure and differentiation, and haplotype analysis

We used STRUCTURE 2.3 software to assess population structure based on simple sequence repeat (SSR) marker data from the 524 plant samples (Falush, Stephens, & Pritchard, 2003; Lawson, Dorp, & Falush, 2018; Pritchard, Stephens, & Donnelly, 2000). Ten standalone runs were designed for each *k* value (from 2 to 12), with the following parameters: burn‐in length, 100,000; run length, 100,000; and admixture model with associated allele frequencies. We calculated the best ∆*k* values by LnP(*D*) (Evanno, Regnaut, & Goudet, 2005; Figure S2 and Table S2a, see Dryad upload). Nucleotide diversity was analyzed using Power Marker V3.25 (Liu & Muse, 2005) and PopGEN32 (http://cc.oulu.fi/~jaspi/popgen/popdown.htm). Genetic diversity coefficients between populations were evaluated using Arlequinver3.1 (http://cmpg.unibe.Ch/software/arlequin3/) and Power Marker V3.25 from Liu and Muse (2005). Neighbor‐joining (NJ) cluster analysis was carried out using Mega6.0 (Tamura, Stecher, Peterson, Filipski, & Kumar, 2013) and Power Marker V3.25. All DNA sequences were translated into Clustal format. ClustalX2 was then used to remove redundant fragments at either end, align sequences, and filter erroneously aligned nucleotides. We then used Multi Domain Analysis in DnaSPv5.0, using Network5.0 to construct the evolutionary network.

### 2.3 Plant growth, RNA isolation, RNA sequencing, and read filtering

Seeds of all plant materials were sown in 24 equal‐size pots (length: 60 cm, width: 30 cm, depth: 25 cm) containing equivalent nutrient soil for two replicates in a greenhouse on November 14, 2016 at the Chinese Academy of Agricultural Sciences, Beijing. All plants were grown under the same conditions (21–23°C, 2.5 L of H_2_O every 2 days). On January 22, 2017, at the 3.5‐leaf stage, the third leaf of each seedling was sampled for RNA extraction from five WR lines (WR16, WR21, WR24, WR162, WR248), five LR (HD, MDL, HMD, WG‐7, XHBD), and five IR cultivars (LG288, JD106, LX15, LX16, NG45). Total RNA was obtained from the leaf tissue using the RNA easy Mini Kit (Qiagen). A NanoDrop2000 spectrophotometer (Nano Drop Technologies) was used to assess the quantity and quality of the RNA. An Agilent 2100 Bioanalyzer (Agilent technologies) was used to evaluate the integrity of the purified RNA based on the RNA integrity number (RIN). High‐quality total RNA was then used for mRNA enrichment and to construct libraries for RNA sequencing. Sequencing was performed using the high‐throughput Illumina HiSeq2500/4000 sequencing platforms. A total of 220,476,506 raw read pairs were obtained, and after quality control, 206,766,127 clean read pairs remained. Of the cleaned reads, 88.67% had an average quality score of Q30 (Simon, Paul, & Wolfgang, 2015) for the 15 rice varieties; Q30 represents a base‐calling error rate of 1 in 1,000.

### 2.4 RNA‐seq data analysis

The data analyses performed included comparative, gene structure, and gene level analyses. Comparative results were sorted, some sequence duplicates due to excessive PCR amplification were removed using SAMtools or Picard, SNPs/indels were checked using GATK software (Broad Institute), and low‐quality results were removed. Based on the predicted gene models according to Cufflinks, alternative splicing (AS) events were sorted and counted using ASprofile software. To compare different gene lengths, different experiments, and different amounts of sequencing data, fragments per kilo base of exon model per million mapped reads (FPKM) was used to evaluate gene expression levels. The union model was used to analyze gene expression levels through HTSeq (http://htseq.readthedocs.io/en/release_0.9.1/). SNPs and differentially expressed genes (DEGs) were analyzed in the 15 samples. DEG‐GO (Gene Ontology, http://www.geneontology.Org/) and DEG‐KEGG (Kyoto Encyclopedia of Genes and Genomes, http://www.Kegg.Jp) analyses were carried out based on the DEGs (Young, Wakefield, Smyth, & Oshlack, 2010) and the KEGG database (Kanehisa et al., 2008).

## 3 RESULTS

### 3.1 Population genetic analysis based on SSR marker data

As seen in Figure 1, the study population of 524 rice lines consisted of 136 WR samples from China and Korea (*H*_e_ = 0.8386), 161 LR samples (*H*_e_ = 0.8094), 185 IR samples (*H*_e_ = 0.7857), and 42 samples of wild rice (*H*_e_ = 0.8791). We compared the genetic diversity and differentiation of WR and the other rice populations. The average values for the genetic diversity indexes were ranked in the order wild rice (0.8791) > WR (0.8386) > LR (0.8094) > IR (0.7857) (Figure 1a; Table S1 see Dryad upload). The average value for the genetic differentiation coefficients was WR/wild rice (0.0806) > WR/IR (0.0558) > WR/LR (0.0236), and the minimum value was between WR and LR (Figure 1b).

**Figure 2:**
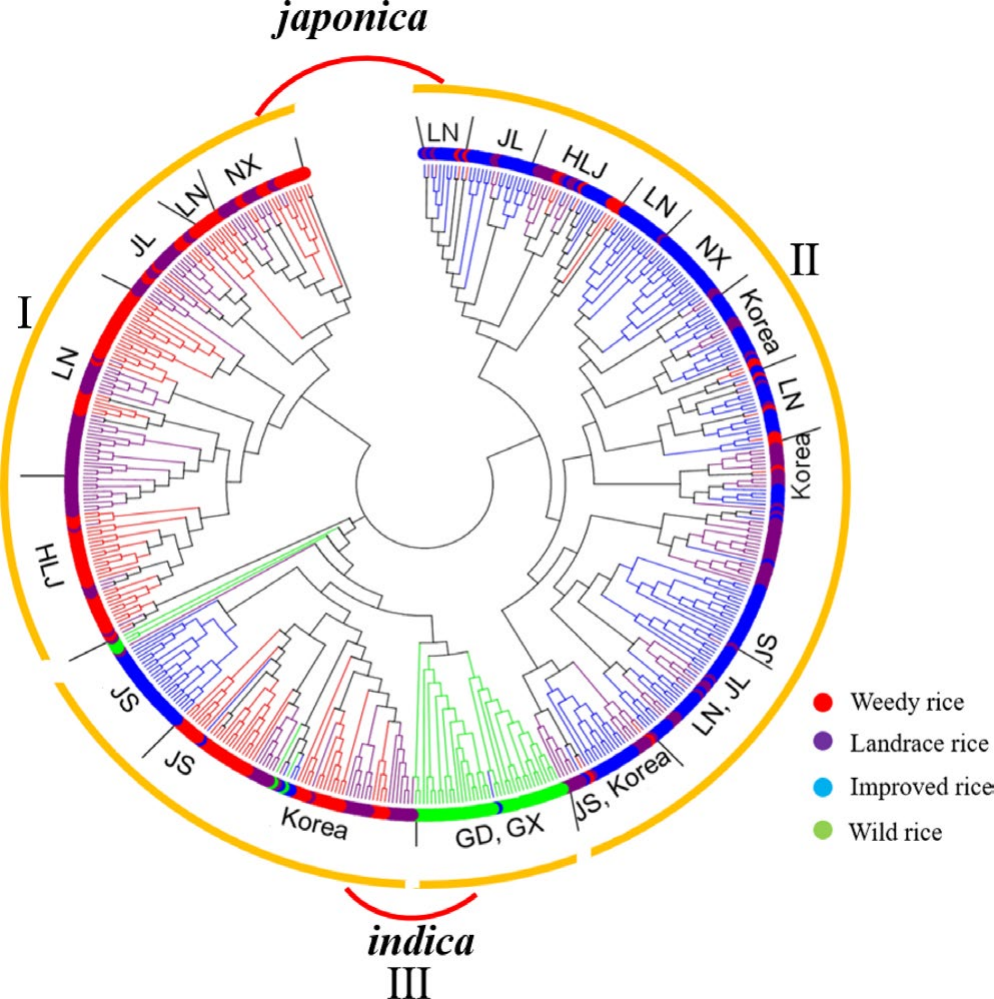
NJ cluster analysis‐based SSR markers for 524 samples. NJ cluster analysis for 524 samples. HLJ, Heilongjiang province in China; JL, Jilin province in China; LN, Liaoning province in China; NX, Ningxia province in China; JS, Jiangsu province in China; GD, Guangdong province in China; GX, Guangxi province in China

**Figure 3:**
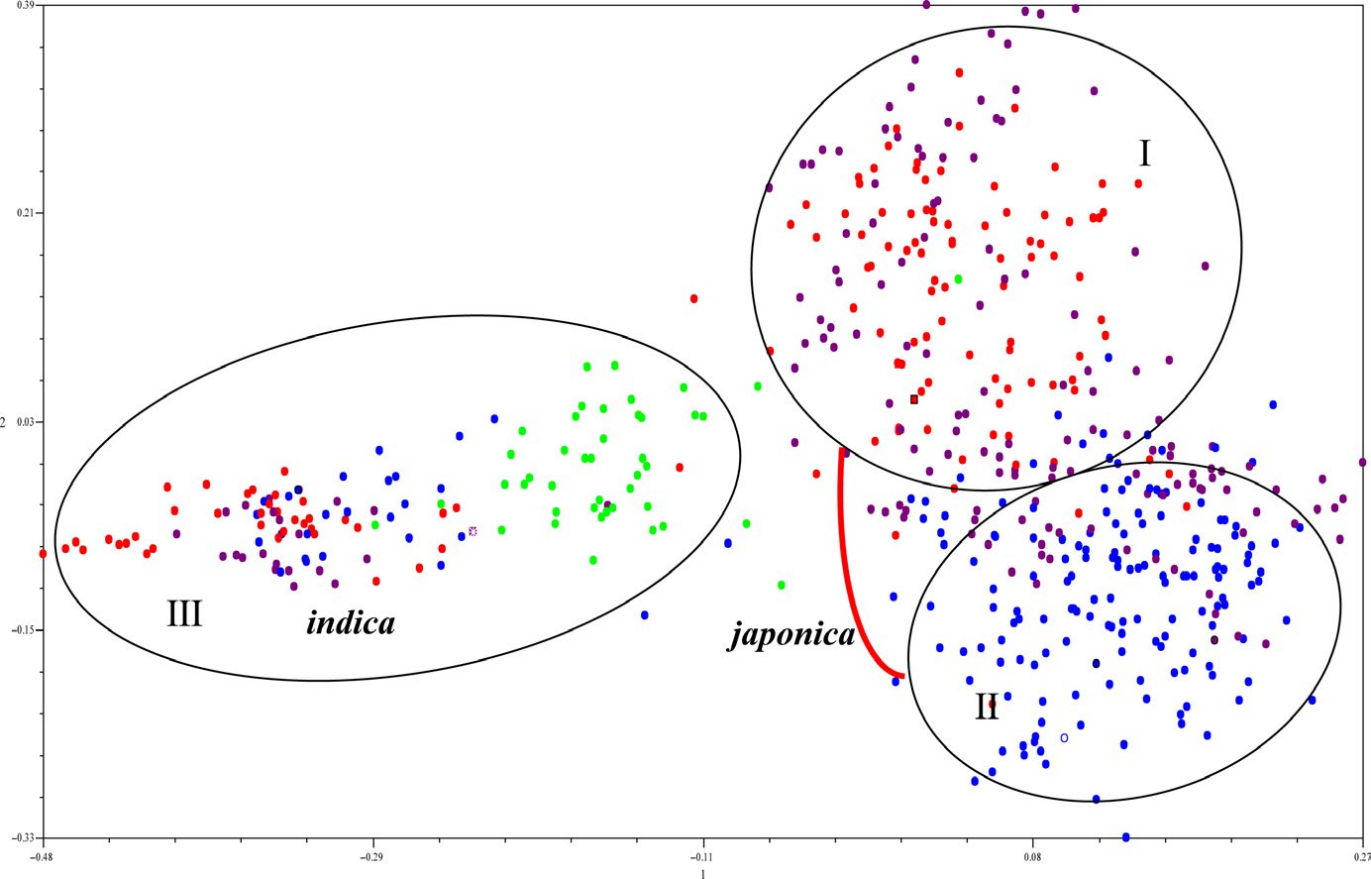
Principal components analysis (PCA) of the 524 genotypes based on 36 pairs SSR markers. Red color represents weedy rice (WR), purple color represents landrace rice (LR), blue color represents improved rice (IR), and green color represents wild rice

To assess the evolutionary relationships between WR and the other populations, we constructed a neighbor‐joining (NJ) tree using data for the 524 samples (Figure 2; Table S1 see Dryad upload). The NJ tree divided our samples into three groups; Group I consists of plants in the *japonica*‐type population, and also includes landrace varieties from HLJ, JL, LN, and NX; Group II also contains *japonica*‐type rice lines and includes IR cultivars from HLJ, JL, LN, and NX; Group III represents the *indica*‐type population, and includes landraces and improved varieties from JS province and Korea and wild rice collections from GD and GX provinces. The PCA analysis (Figure 3) gave the same result as that shown Figure 2, in which the 524 samples were divided into three groups (I, II, III).

**Figure 4:**
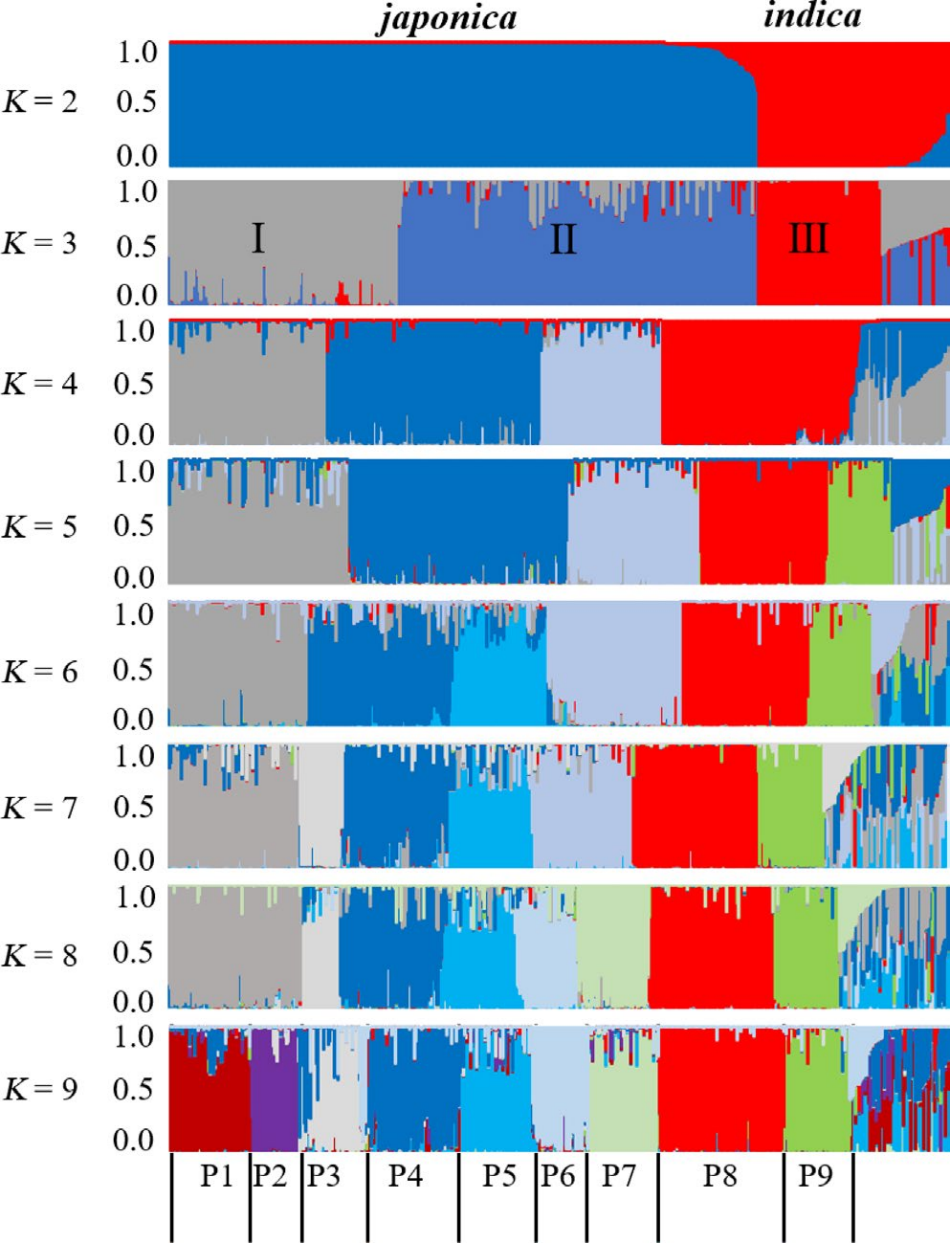
Population structure analysis for 524 samples. HLJ, Heilongjiang province in China; JL, Jilin province in China; LN, Liaoning province in China; NX, Ningxia province in China; JS, Jiangsu province in China; GD, Guangdong province in China; GX, Guangxi province in China. P1 population represents weedy rice (WR) from HLJ, JL, and LN province, P2 groups represents LR from HLJ, JL, and LN province, P3 represents WR and LR from NX province, P4 represents improved rice (IR) from HLJ, JL, and LN provinces, P5 represents IR from NX province, P6 represented *japonica*‐type IR and *japonica*‐type LR from JS province and Korea, P7 represents *indica*‐IR from JS, P8 represents the mixed WR from JS and Korea and *indica*‐type LR from JS, P9 represents wild rice

To further study the population structure of the 524 rice varieties, a Bayesian analysis was performed using STRUCTURE V. 2.3 (Falush et al., 2003; Figure 4; Table S1 see Dryad upload). *K* was set from 2 to 12.

When *K* = 2, the 524 lines were divided into two groups (*japonica* and *indica*; Figure 4; Figure S2 see Dryad upload), and at *K* = 3, the population was divided into three subpopulations (I, II, III) which is consistent with the results from our NJ and PCA analyses. At *K* = 9, the WR lines (P1 marked dark red) from HLJ, JL, and LN provinces were separated from the HLJ, JL, and LN landraces (P2 marked purple), suggesting that the WR population is different from the LR groups. However, the WR lines from NX were still mixed with the NX landraces (P3), which suggests a closer genetic relationship between NX WR and landraces compared to that between WR and the landraces from HLJ, LN, and JL provinces. Weedy rice collections from JS and Korea (P8 marked red) were always mixed with the *indica* landraces from JS. For K values from 2 to 9, there was no evidence that WR originated directly from wild rice.

The NJ, PCA, and population structure analyses indicate that *japonica‐*type WR is not closely related to wild rice. In contrast to *japonica* WR, the *indica‐*type WR lines showed a more mixed pattern, and they grouped with the *indica‐*type landraces and the IR cultivars and clustered with the wild rice collections on the group III branch. This indicates that the origins of *indica*‐type WR are complicated, and that local *indica*‐type rice and wild rice all contributed to the genomes of *indica*‐type WR.

### 3.2 Haplotype analysis of domestication genes

**Figure 5:**
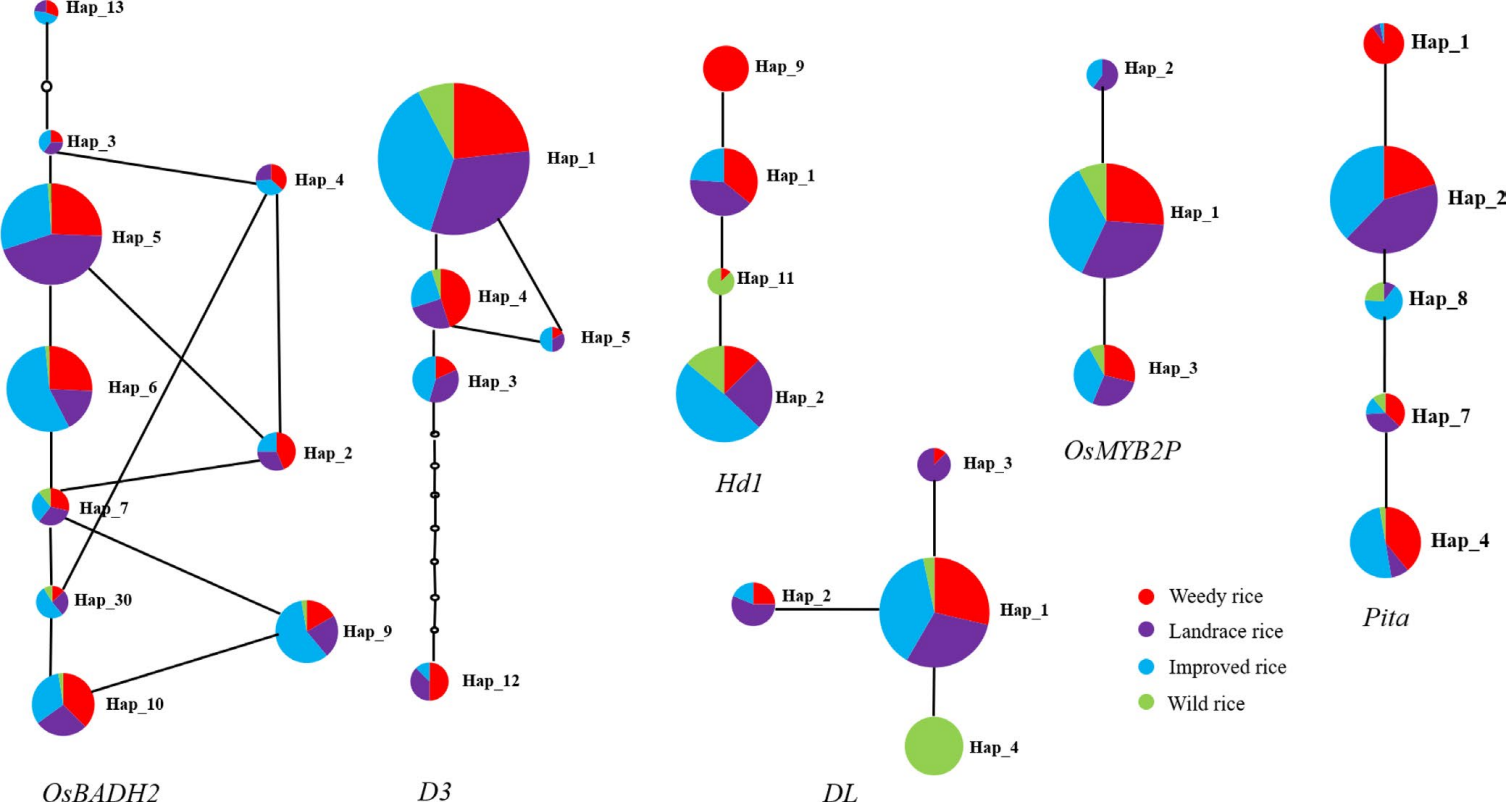
TCS evolutionary network based on six gene haplotypes. Red color represents weedy rice (WR) populations, Violet color represents landrace rice (LR) populations, Blue color represents improved rice (IR) populations, and Green color represents wild rice populations. The size of the circle represents the frequency of the haplotype, the lines between haplotypes represent the mutation. The hollow circle represents missing haplotypes

To study the evolution of the haplotypes, we amplified fragments of the six unlinked genes *Hd1*, *D3*, *DL*, *OsMYB2P*, *Pita*, and *OsBADH2* from the 524 rice accessions. The lengths of the amplified fragments were 491, 780, 597, 601, 891, and 727 bp, respectively. Indels were not included in the analyses. The main haplotypes were calculated, and a TCS evolution network was constructed based on the major haplotypes for the six loci (Figure 5). All critical haplotypes for each gene were displayed on the network branches. Hap_5 haplotype for *OsBADH2* had the highest frequency compared with other haplotypes and was shared by WR, LR, and IR cultivars, while Hap_7 contained WR, landraces, IR, and wild rice and is one of the oldest haplotypes with the most branches. For the *D3* gene, Hap_1 contained WR, LR, IR, and wild rice with the highest frequency. For *Hd1*, Hap_2 contained WR, LR, IR, and wild rice with the highest frequency, while WR was the only type represented in Hap_9. For *DL*, Hap_1 contained WR, LR, IR, and wild rice with the highest frequency and the most branches. For *OsMYB2P*, Hap_1 contained WR, IR, LR, and wild rice with the highest frequency. For *Pita*, Hap_2 contained WR and LR with the highest frequency.

### 3.3 Comparative transcriptomic analysis of 15 rice samples

**Figure 6:**
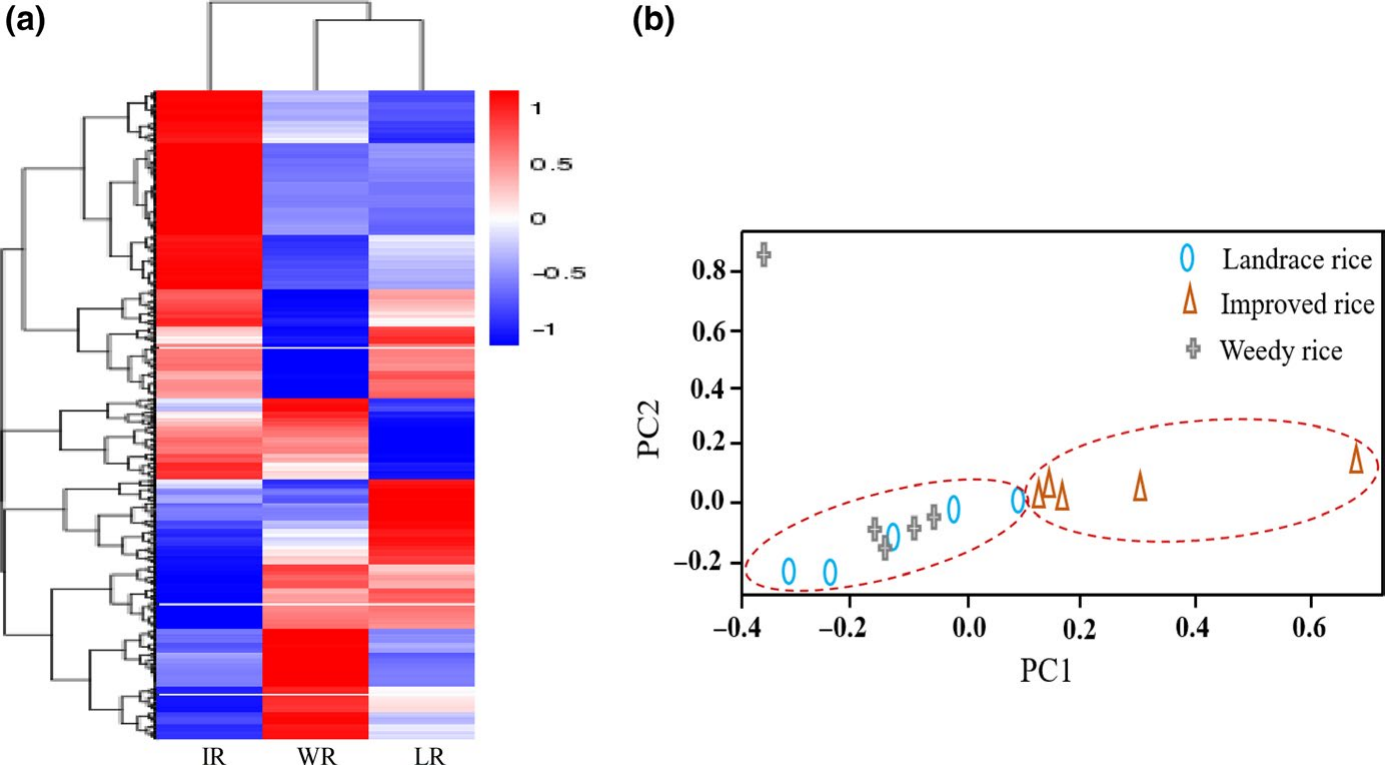
NJ cluster and PCA analysis based on SNPs from five WR varieties, five landraces and IR varieties (a) NJ cluster analysis based on the SNPs, WR represents weedy rice, LR represents landrace rice, IR represents improved rice. (b) PCA analysis based on SNPs from IR, WR, and LR. The oval represents the LR, the triangle represents IR, and the plus sign represents WR samples

RNA‐seq was performed on representative varieties; five WR lines (WR16, WR21, WR24, WR162, WR248), five LR (XHBD, MDL, HMD, HD, and WG7), and five IR cultivars (NG45, JD106, LX15, LX16, and LG288) from HLJ, JL, LN, and NX provinces. A total of 84,278 SNPs were detected in all samples comparing to the “Nipponbare” (*O. sativa* ssp. *japonica*) control. Based on these SNPs, a phylogenetic tree of the 15 RNA samples was constructed using the neighbor‐joining (NJ) method (Figure 6a; Table S2 see Dryad upload). The WR collections clustered with the LR and IR, which is consistent with a genetic analysis based on neutral markers. The results of a PCA analysis (Figure 6b, Table S2 see Dryad upload) showed that the WR lines are distributed within an area defined by the landraces, and the genetic distance between WR and the landraces indicates that they are closer to each other than either is to the IR cultivars, which is consistent with the results based on the neutral markers.

**Figure 7:**
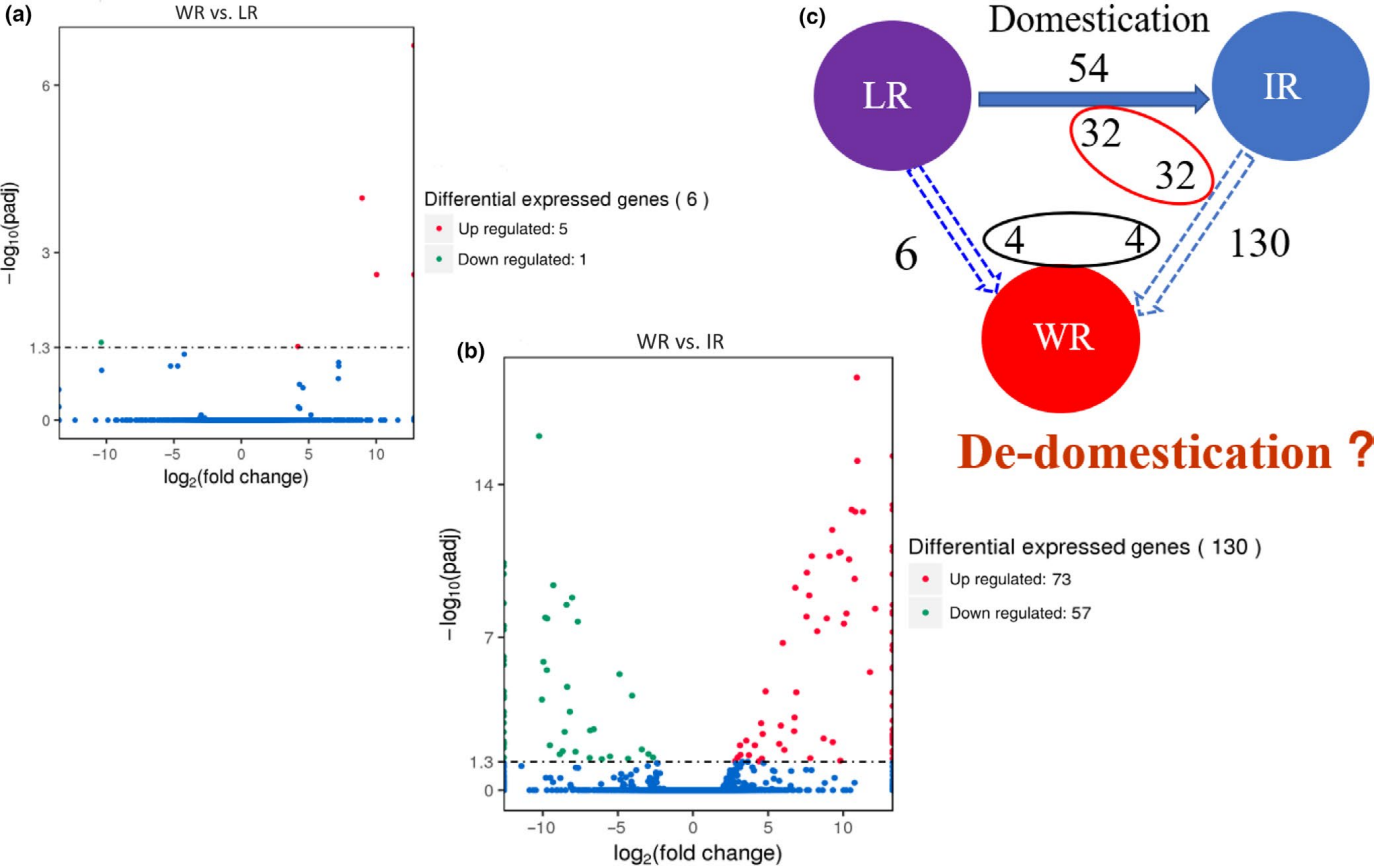
The analysis of differentially expressed genes between WR, LR, and IR. (a) The number of differentially expressed gene between WR and LR. (b) The number of differentially expressed genes between WR and IR. (c) Codifferentially expressed gene analysis between LR, IR, and WR. WR represents weedy rice, IR represents improved rice, and LR represents landrace rice

We analyzed the genes that showed differential expression levels between the WR, LR, and IR groups (Figure 7). Between the weedy and landrace groups, gene expression was found to be significantly different in six genes, with up‐regulation of five genes and down‐regulation of one gene (Figure 7a, Table S3 see Dryad upload). However, between WR and IR, gene expression was significantly different in 130 genes; 73 genes were up‐regulated and 57 were down‐regulated (Figure 7b, Table S4 see Dryad upload). Between the landraces and IR cultivar, gene expression was significantly different in 54 genes, with 23 genes up‐regulated and 31 down‐regulated (Table S5 see Dryad upload). In summary, there were fewer differentially expressed genes (DEGs) between the weedy and LR groups than between the weedy and IR groups under the same experimental conditions, which explains why the WR collections clustered with LR in the PCA. The DEGs that were shared between the different evolutionary or domestication processes were analyzed by comparing the process going from the LR to IR, and the process going from the IR to WR. We found that 32 DEGs were shared between the process going from the LR to IR and the process going from IR to WR (Figure 7c).

**Figure 8:**
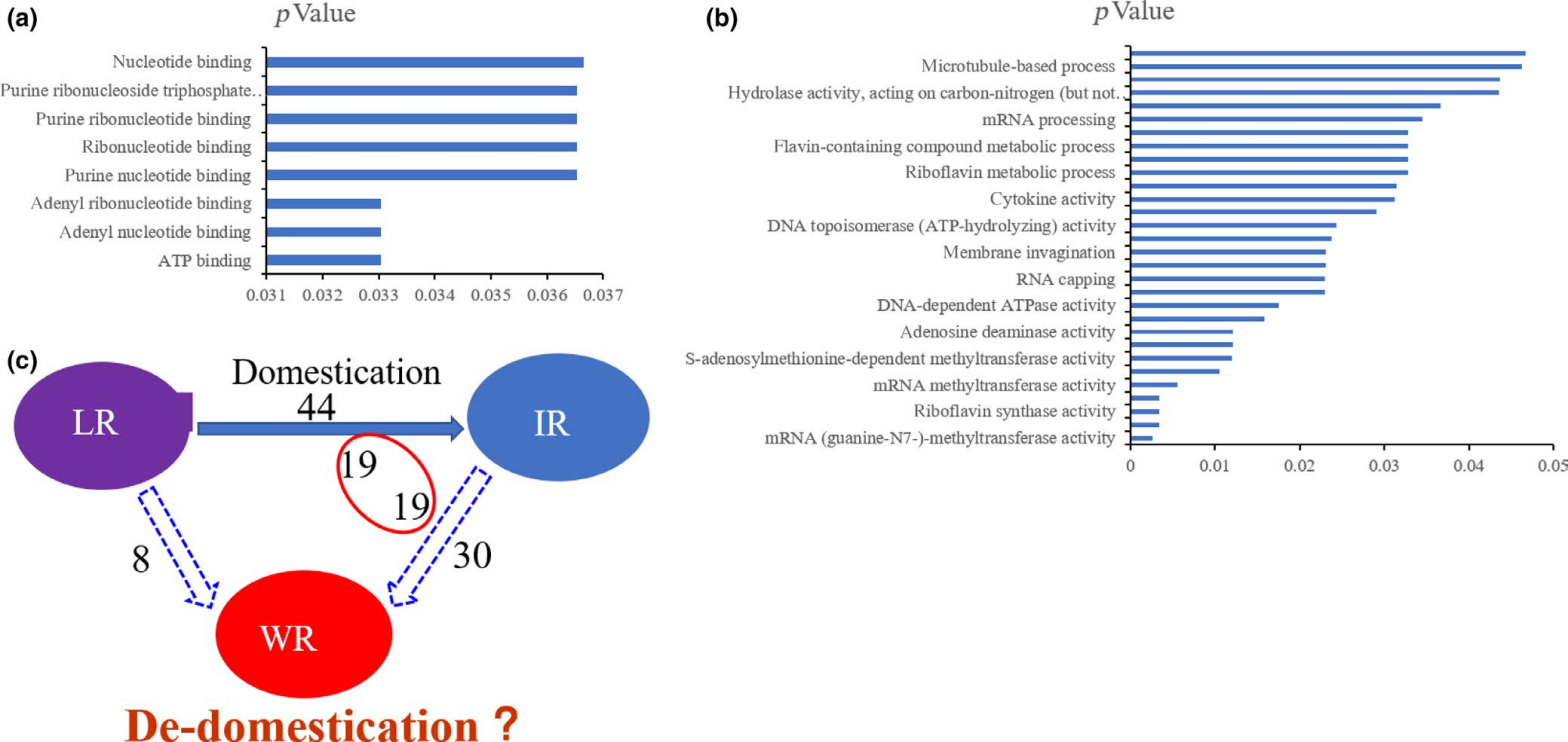
The functional analysis of DEGs between WR, LR, and IR. (a) The enrichment terms of differentially expressed genes between WR and LR. (b) The enrichment terms of differentially expressed genes between WR and IR. (c) The coenrichment terms between WR, IR, and LR. WR represents weedy rice, IR represents improved rice, LR represents landrace rice (*p*‐value < .05)

To explore the biological functions of the DEGs, a functional enrichment analysis was conducted to examine the gene ontology (GO) terms between the WR and landrace populations. In all, eight GO terms were significant (*p* < .05) and were in the primary GO category of “molecular function” (Figure 8a,c; Table S6 see Dryad upload). Between WR and IR, 30 GO terms were significantly enriched (*p* < .05) and were located in the three major GO categories: “biological process,” “molecular function,” and “cellular component” (Figure 8b,c; Table S7 see Dryad upload). Between the LR and the IR cultivars, 44 GO terms were found to be significantly enriched (*p* < .05) in the three major GO categories (Table S8 see Dryad upload). Therefore, in the evolution of the three rice populations, molecular epigenetic inheritance can play critical roles. The enriched GO terms of the DEGs shared between the different domestication processes were analyzed by comparing the processes going from the landraces to IR and from IR to WR. We found that 19 terms were shared between these comparisons. When comparing the evolutionary or domestication processes going from the landraces to WR, we found eight shared GO terms (Figure 8c).

## 4 DISCUSSION

Weedy rice grows as a weed in cultivated rice fields and negatively affects rice production worldwide. Recent studies have indicated that WR plants growing in different regions around the world appear to have multiple origins (He et al., 2017; Li et al., 2017; Qiu et al., 2014, 2017; Sun et al., 2013, 2019). Weedy rice can lead to a severe reduction in the yields of cultivated rice (*O. sativa*) in many rice‐growing regions of the world. Understanding the genetic diversity and population structures of WR will help researchers effectively trace its origins and distribution patterns in some regions.

### 4.1 Origins of weedy rice based on neutral markers and domestication genes

As shown in Figure 1, we found that the greatest genetic diversity was in the wild rice population (*H*_e_ = 0.8791) and the lowest genetic diversity was in the improved the rice population (*H*_e_ = 0.7857) compared to the other two rice populations (WR = 0.8386; LR = 0.8094). The genetic diversity of the WR population (*H*_e_ = 0.8386) falls between that of the wild rice and landrace populations. He et al. (2014) studied genetic diversity in 21 WR populations from Sri Lanka and found an overall genetic diversity index of *H*_e_ = 0.62; the higher genetic diversity index (*H*_e_ = 0.8386) of the WR population in our study suggests that the population structures of WR populations from China and Korea are more complicated compared with those from Sri Lanka. In addition, the high level of genetic diversity within WR populations suggests that the origin of the WR collections included in our study is more diversified.

Phylogenetic (NJ, Figure 2), PCA (Figure 3), and STRUCTURE analyses (Figure 4) all indicate that the WR collections included in our study are divided into two populations, *indica*‐type and *japonica*‐type. Further analysis showed that the WR plants collected from HLJ, JL, and LN provinces, located in Northeast China, clustered with the *japonica*‐type landraces from HLJ, JL, and LN provinces, suggesting that the WR found in these three provinces represents a possible de‐domestication origin from cultivated rice (IR). The *japonica*‐type WR collections from NX province always grouped with landraces grown in NX, indicating that WR and LR from NX share a close genetic relationship. Long‐term direct‐seeded rice farming in the NX region could be a major cause of introgression of landrace genes into the genome of WR in NX province. The WR samples collected from JS province clustered with the *indica*‐type LR from JS province, suggesting that WR from JS province originated from hybridization between *indica* varieties. The Korean WR population that we investigated in this study was a control in the analysis, and the results showed that the Korean WR collections can be divided two populations, an *indica*‐type population that clusters with the *indica*‐type landraces from JS province, and a *japonica*‐type population that clusters with the *japonica*‐type IR cultivars from Northeast China. Therefore, the origins of WR from China are diverse, and multiple evolutionary models drove the development of WR in different regions of China and contributed to the high level of genetic diversity (*H*_e_ = 0.8386).

It is clear from our study that the origins of WR in China are complex, and that WR arose multiple times from the de‐domestication of IR (HLJ, JL, and LN), and from *indica*/*indica* hybridization (JS) as well as from gene introgression or de‐domestication from LR (NX). China's history of rapid population growth and internal migration patterns, as well as the geological and climatic diversity, may have driven the complex evolution of WR.

The TCS evolutionary network (Figure 5), based on the major haplotypes of six domestication genes, showed that Hap5 for *OsBADH2* and Hap_2 for *Pita* had the highest frequencies and are shared by WR, LR, and IR cultivars which suggests that the haplotypes found in WR originated from cultivated rice (landraces and IR). Hap_1 for *D3*, Hap_2 for *Hd1*, Hap_1 for *DL,* and Hap_1 for *OsMYB2P* are all shared by WR collections, IR cultivars, LR, and wild rice at high frequencies, suggesting that the gene haplotypes of some WR samples have their origins in wild or cultivated rice. Reagon et al. (2010) inferred the origins of U.S. WR based on single‐nucleotide polymorphisms (SNPs) by comparisons with domesticated and wild rice samples. Based on our haplotype analysis, WR lines from China also show complex origins of gene haplotypes. The haplotypes of IR cultivars, LR, and even wild rice could have contributed some haplotypes to WR.

### 4.2 Origins of weedy rice based on comparative transcriptomics

In the history of crop domestication, artificial and natural selection have greatly changed crop growth patterns, physiology, and life history. Environmental and artificial selection have played critical roles in this process, and some evolutionary footprints have been retained and passed on to offspring through crop genomic DNA or RNA expression. In our study, leaf tissues from representatives of different rice groups or subpopulations (five accessions each of WR, IR, and LR) from HLJ, JL, and LN provinces were sampled at the 3.5‐leaf stage for RNA isolation. RNA‐seq was performed on these samples, and the differentially expressed genes (DEGs) were identified between WR, IR, and the landraces (Figure 7). Between the landraces and IR cultivars, we found 54 DEGs; 130 DEGs were present between WR and IR; and only six DEGs were found in the comparison of WR and LR (Figure 7a,b, Tables S3–S5 see Dryad upload). These results could explain why the WR collections always clustered with the local landrace populations.

Further analysis showed that 32 DEGs were shared by the improved cultivars and LR groups as well as between the improved cultivars and the WR groups (Figure 7c). These shared DEGs suggest that the evolution from landrace to IR and the evolution from IR to WR are linked. The evolutionary process going from LR to IR is one part of the domestication history of rice, and therefore, the evolutionary process going from IR to WR should be only one part of WR evolution. In the two processes (from landrace to IR, and from IR to WR), IR is the common endpoint, which leads us to speculate that IR cultivars are one of endpoints in the evolution of WR.

To study the function of the DEGs in the process or recent rice evolution, we performed GO‐term analysis based on the DEGs (Figure 8 and Tables S6–S8). Between the LR and the IR cultivars, 44 terms were found to be enriched; 30 terms were enriched between WR and IR, but only eight terms were enriched between WR and the landraces (Figure 8). Our analysis showed that the majority of these enriched biological process terms were due to epigenetic modification, such as methylation and binding, suggesting that epigenetics factors significantly in the history of rice domestication. In addition, we analyzed the shared domestication processes and identified 19 terms that were shared by the processes going from the landraces to IR and from IR to WR (Figure 8c). These results show that the trajectory is from IR to WR or from the LR to WR in the evolutionary history of WR, and that de‐domestication from IR cultivars may have played a critical role in the evolution of WR in HLJ, JL, and LN provinces.

## CONFLICT OF INTERESTS

On behalf of all authors, the corresponding author states that there is no conflict of interest.

## AUTHOR CONTRIBUTIONS

B. H designed the research, performed the research, conducted field work, data analysis, collection, or interpretation, and wrote the manuscript; X‐D. M helped for field work; D. C helped for field work; Y‐J. W helped for field work; L‐Y. G helped for field work; G‐L. C helped for field work; H. Z guided experiments and manuscript revision; H‐J. K designed the research and manuscript revision; and L‐Z. H designed the research, conducted field work and manuscript revision.

## Data Availability

Data (Figures S1 and S2 and Tables S1–S8) can be found on Dryad, http://doi.org/10.5061/dryad.sqv9s4n0h (Han et al., 2019).
